# HCMV Displays a Unique Transcriptome of Immunomodulatory Genes in Primary Monocyte-Derived Cell Types

**DOI:** 10.1371/journal.pone.0164843

**Published:** 2016-10-19

**Authors:** Ellen Van Damme, Kim Thys, Marianne Tuefferd, Carl Van Hove, Jeroen Aerssens, Marnix Van Loock

**Affiliations:** 1 Infectious Diseases, Janssen Pharmaceutica NV, Beerse, Belgium; 2 Discovery Sciences, Janssen Pharmaceutica NV, Beerse, Belgium; University of St Andrews, UNITED KINGDOM

## Abstract

Human cytomegalovirus (HCMV) is a betaherpesvirus which rarely presents problems in healthy individuals, yet may result in severe morbidity in immunocompromised patients and in immune-naïve neonates. HCMV has a large 235 kb genome with a coding capacity of at least 165 open reading frames (ORFs). This large genome allows complex gene regulation resulting in different sets of transcripts during lytic and latent infection. While latent virus mainly resides within monocytes and CD34^+^ progenitor cells, reactivation to lytic infection is driven by differentiation towards terminally differentiated myeloid dendritic cells and macrophages. Consequently, it has been suggested that macrophages and dendritic cells contribute to viral spread *in vivo*. Thus far only limited knowledge is available on the expression of HCMV genes in terminally differentiated myeloid primary cells and whether or not the virus exhibits a different set of lytic genes in primary cells compared with lytic infection in NHDF fibroblasts. To address these questions, we used Illumina next generation sequencing to determine the HCMV transcriptome in macrophages and dendritic cells during lytic infection and compared it to the transcriptome in NHDF fibroblasts. Here, we demonstrate unique expression profiles in macrophages and dendritic cells which significantly differ from the transcriptome in fibroblasts mainly by modulating the expression of viral transcripts involved in immune modulation, cell tropism and viral spread. In a head to head comparison between macrophages and dendritic cells, we observed that factors involved in viral spread and virion composition are differentially regulated suggesting that the plasticity of the virion facilitates the infection of surrounding cells. Taken together, this study provides the full transcript expression analysis of lytic HCMV genes in monocyte-derived type 1 and type 2 macrophages as well as in monocyte-derived dendritic cells. Thereby underlining the potential of HCMV to adapt to or influence different cellular environments to promote its own survival.

## Introduction

Human cytomegalovirus (HCMV) is a ubiquitous species-specific betaherpesvirus that infects between 60–100% of the population depending on age, gender, demographics and socio-economic status [1]. The 235kb HCMV genome is characterized by unique long (UL) and unique short (US) regions, each flanked by terminal (TRL and TRS) and internal (IRL and IRS) inverted repeats [2].

Transcription of the HCMV genome is complexly regulated and different sets of transcripts are expressed during productive and latent infection. During latency, the major immediate-early (IE) promotor, which drives IE gene expression, is epigenetically blocked. However, early studies in experimental latency models detected several sense and antisense CMV latency-associated transcripts (CTLs) expressed from the major immediate-early region of the genome, although these CTLs are controversial [3–5]. Since then four transcripts i.e. UL81ast/LUNA [6–10], UL111A [10–12], UL138 [8, 10, 13, 14] and, depending on the virus used and the presence of GATA transcription binding sites, UL144 [15] were characterized thoroughly as latency-associated transcripts. Alternatively, during productive infection, the full array of genes is expressed and gene expression follows a temporal cascade starting with immediate-early gene expression, succeeded by early and late gene expression [16–22]. The transcriptome consists of both polyadenylated protein coding and polyadenylated non-protein coding RNAs. The latter RNA species is comprised of four different long non-coding RNAs (lnc RNAs) which do not overlap with any of the protein coding regions. These four lnc RNAs have been reported to be responsible for 65% of the total polyadenylated RNA pool [23, 24]. In addition, HCMV also expresses non-coding RNA in the form of anti-sense transcripts which often overlap with protein coding genes, and microRNAs which have been attributed a regulatory function [23–25]. Currently, the transcriptome of HCMV has not been entirely unraveled. It has long been assumed that the genome of clinical isolates contained about 170 genes [23, 24]. However, recently a new study revealed an unexpected complexity when Stern-Ginossar et al. identified hundreds of new ORFs through ribosome profiling and transcript analysis in lytically infected fibroblasts [26]. Currently, the functions of these newly discovered transcripts remain largely unknown.

Only recently, transcriptomics technology platforms have been used to study the influence of HCMV infection on host and viral gene expression in different cell lines and cell types. Towler et al. showed that, depending on the cell type, the temporal expression of viral genes varies; but also that, compared to fibroblasts, the transcriptome in epithelial cells and astrocyte cell lines is differentially regulated [27].

CD34^+^ progenitor cells and CD14^+^ monocytes are currently the only confirmed sites of latency [28, 29]. Upon differentiation to macrophages or dendritic cells, the virus reactivates and lytic replication is enabled, possibly promoting viral spread after reactivation [17, 30]. The transcriptome in latently infected CD34^+^ progenitor cells and CD14^+^ monocytes is slowly being unraveled [8, 10, 12]. However, only one study aimed at delineating the HCMV transcriptome in permissive myeloid cells [31]. It remains unclear how the viral transcriptome adapts to infection in macrophages (MΦs) and dendritic cells (DCs) and if the virus shows a different transcriptional profile in pro-inflammatory DCs or macrophages type 1 (MΦ1) or anti-inflammatory macrophages type 2 (MΦ2). Therefore, we used Illumina next generation sequencing to characterize how HCMV modulates gene expression specifically in terminally differentiated monocyte-derived MΦ1, MΦ2 and DCs in comparison to fibroblasts.

The data reported here is, to our knowledge, the first next generation sequencing transcriptome study investigating HCMV transcription in cell types which are relevant for *in vivo* viral spread. We show that HCMV follows a specific transcriptional program in primary cells in which genes involved in virion composition, cell tropism or cell-specific replication, viral spread and immunomodulation are expressed at different levels compared to fibroblasts. This not only highlights the potential of HCMV to adapt to or influence different cellular environments to promote its own survival. This also reflects the significant differences in gene expression between cell types used in the laboratory environment and in primary cell types.

## Materials and Methods

### Ethics statement

Peripheral blood mononuclear cells (PBMCs) and primary monocytes were isolated from whole blood. Blood was obtained via the Antwerp Blood Transfusion Center of the Red Cross (www.redcross.be). Donors gave written consent for their samples to be used for scientific research.

### Cell lines

ARPE-19 cells (CRL-2302, ATCC) and neonatal NHDF fibroblasts (CC-2509, Lonza) were maintained and propagated in DMEM:F12 with L-glutamine (Biowhittaker) and MEM (Life Technologies) respectively, containing 10% heat-inactivated FCS (HI-FCS; Life Technologies) and 0.04% gentamicine. All infection experiments were done in 24-well plates (Costar).

### Culturing of clinical isolates TB40/E

High epithelial tropic stocks of the HCMV BAC4-TB40/E [32] were generated in ARPE-19 cells. In brief, ARPE-19 cells were seeded 24 hours before infection to reach 40–60% confluence at the time of infection (3.5 x 10^6^ cells/T175). Incubation with TB40/E at MOI 0.02 was done for 3 hours at room temperature on a rocking platform. The virus was harvested until the cells detached from the flasks. Harvests were spun down for 10 minutes at 600x*g* to remove cellular debris. All harvests were titrated (see below) and only the 6 harvests with the highest titers were used for concentration. Viral stocks were prepared by centrifugation (1 hour at 3000x*g*) and ultracentrifugation (1 hour at 5800x*g*). Concentrated virus was resuspended in 5 ml MEM/10% FCS and kept at -80°C for long term storage.

### Virus titration using immediate-early protein immunofluorescent staining

Virus yield was determined on NHDF and ARPE-19 cells by an immune-fluorescence assay adapted from Chou et al. [33]. NHDF cells and ARPE-19 were used to determine TB40/E titers or epithelial tropism, respectively. In brief, 15000 cells (NHDF or ARPE-19 cells) per well were seeded in a 96-well plate in 75 μl MEM/2% HI-FCS or DMEM:F12/2% HI-FCS. Then, quadruplicates of a ten-fold dilution series of the virus was added to the cells (25μl input). Three days post infection, the viral replication was determined after staining for immediate-early antigen expression in an immune-fluorescence assay (see below). Wells were scored positive if they contained at least one colony of 2–5 IE-positive cells. After scoring, the TCID_50_/ml was calculated based on the Spearman-Kärber method.

### Immediate-early protein staining

The medium of infected cells was removed and the cells were washed once with phosphate buffered saline (PBS). Subsequently, the cells were fixed using 100% ice-cold methanol (10 minutes at -20°C). To remove residual methanol, the cells were washed twice with PBS. Staining with the primary antibody was carried out for 20 minutes at room temperature (RT) with an anti-immediate-early antigen antibody (anti-I.E.A., 11–003, Argene) diluted 1/100 in PBST (PBS containing 0.05% tween-20). Prior to incubation with anti-mouse antibody conjugated to Alexa Fluor 488 (Alexa Fluor 488 goat anti-mouse IgG, A-11001, Life Technologies), the cells were washed twice with PBS. After 20 minutes incubation at RT, the secondary antibody was removed and the cells were washed twice with PBS. Afterwards, the cells were stored at 4°C in 200μl PBS until readout.

### Isolation of primary CD14+ monocytes and differentiation to HCMV permissive cell types

Peripheral blood mononuclear cells (PBMCs) were isolated from buffy coats using ficoll-paque. In brief, 60 ml blood was added to 100 ml ice-cold PBS. Twenty-five ml diluted blood was carefully applied on 15 ml ficoll-paque. After centrifugation (800x*g*, 20 minutes, no brake), the PBMC fraction was collected and divided over four 50 ml tubes. Each tube was filled with ice-cold PBS to a total volume of 50 ml and the cells were spun down for 10 minutes at 300x*g*. This washing procedure was repeated twice. The total number of PBMCs was determined by manual counting under the light microscope with a counting chamber. Subsequently, monocytes were isolated using magnetic CD14 microbeads (Miltenyi, 130-050-201) as instructed by the manufacturer.

The monocytes were counted manually under a light microscope using a counting chamber, resuspended at 1.5 x 10^6^ cells/ml and 1.5 x 10^6^ cells were seeded per well in a 24-well plate (tissue treated, Costar). The CD14^+^ monocytes were allowed to adhere for 3 hours at 37°C/5% CO_2_. After attachment, the PBS was removed and 200μl X-Vivo-15 medium (Lonza) without supplements was added and the cells were left overnight at 37°C/5% CO_2_. Subsequently, monocytes were differentiated to monocyte-derived dendritic cells (DC), monocyte-derived type 1 macrophages (MΦ1) and monocytes-derived type 2 macrophages (MΦ2) in X-Vivo-15 medium containing 2.5mM L-glutamine and 10% HI-FCS supplemented with 100ng/ml GMCSF and 100ng/ml IL4 (adapted from [34, 35]), 100ng/ml GMCSF or 100ng/ml MCSF (adapted from [34]), respectively, for 7 days (all cytokines from Peprotech). The medium was refreshed on day 4 after the start of differentiation. Activation of terminally differentiated cells was done by adding 500mg/ml LPS (Sigma).

On day 8, the cells were infected for 3 hours at room temperature with TB40/E at MOI 5 in 0.5 ml X-Vivo-15 medium containing 2.5mM L-glutamine and 10% HI-FCS. After infection, the virus inoculum was removed and 1 ml fresh medium without cytokines was added. The cells were incubated for 72 hours at 37°C/5% CO_2_ before samples were harvested.

### FACS analysis of surface markers on monocyte-derived cell types

Monocytes were seeded in a 96-well plate at 3x10^5^ cells per well and differentiated to MΦ1, MΦ2 and DCs as described above. After 7 days, the cells were challenged with 500ng/ml LPS (Sigma) and surface markers were determined. Therefore, the cells were detached from the plate using 5mM EDTA (20 minutes at 37°C). The cells were spun down at 300x*g*, the EDTA was discarded and the cells were incubated with a mix of anti-CD14-APC-Cy7, anti-CD209-PerCP-Cy5.5, anti-CD163-PE and anti-CD80-PE-Cy7 (5μl of each antibody, all antibodies from BD). The antibodies were incubated for 20 minutes at 4°C. After incubation, 200μl PBS was added and the cells were spun down at 300x*g*. The supernatant was discarded, 200μl PBS was added once more and the cells were collected by centrifugation. Readout was done on a BD FACS CANTO II and subsequent analysis were done using BD DIVA software. Plots were made in Sigmaplot (Systat Software).

### RNA sequencing using the Illumina next generation sequencing platform

RNA samples were prepared from cells obtained from five 24-wells using a Qiagen RNeasy Plus kit with an additional on-column DNase digestion step according to the manufacturer’s instructions. RNA and DNA concentrations were measured using a ND-1000 spectrophotometer (Nanodrop).

RNA samples were processed for Illumina sequencing using the TruSeq stranded mRNA Library Prep kit (Illumina) according to manufacturer’s instructions. In brief, the workflow involved purifying the poly-A containing mRNA molecules using poly-T oligo-attached magnetic beads. Following purification, the mRNA was fragmented into small pieces using divalent cations at elevated temperature. The cleaved RNA fragments were copied into first strand cDNA using reverse transcriptase and random primers. This was followed by second strand cDNA synthesis using DNA Polymerase I and RNase H. These strand-specific cDNA fragments went through an end repair process to a single ‘A’ base, followed by ligation of the adapters. The products were purified and enriched with PCR to create the final cDNA library. Finally, samples were pooled and sequenced for 147 cycles on a GAIIx (Illumina) using the TruSeq SBS Kit v5 kit (Illumina). The raw signal was processed into individual sequencing reads per sample with the Illumina software (Casava 1.8.2).

All sequence reads per sample were aligned versus the publicly available reference genome for TB40/E (acc. No. EF999921; 2008). This reference genome is annotated with 168 coding sequences (cds) and was manually updated with 9 additional coding sequences from accession number KF297339 (2013). In addition, also four long non-coding RNA’s (RNA1.2, RNA2.7, RNA4.9 and RNA 5.0) were added from accession number KF297339 (2013) [24]. In this study, the TB40/E-BAC4 was used which misses US1-US6 compared to the wild type virus [32], for the analysis the lack of this region was taken into account.

Mapping of the sequence reads and calculating the total read counts per cds were performed using the RNA-seq analysis module of CLC bio’s CLC Genomics Workbench using default parameters. All raw data was uploaded to a public repository (http://www.ebi.ac.uk/ena/data/view/PRJEB15199).

For the statistical analysis, the Bioconductor suite was used [36]. Count data were preprocessed (normalized and variance stabilized transformed for visualization purposes) using the DESeq package [37]. For each individual transcript, differential expression between cell types was performed from normalized counts using a generalized linear model hypothesizing a negative binomial distribution. Individual p-values were adjusted for multiple testing with the Benjamini-Hochberg procedure [38]. Finally, a pathway enrichment analysis was performed using the MLP algorithm [39, 40].

## Results

### HCMV infection of monocyte-derived cell types and percentage HCMV reads

**Fig 1:**
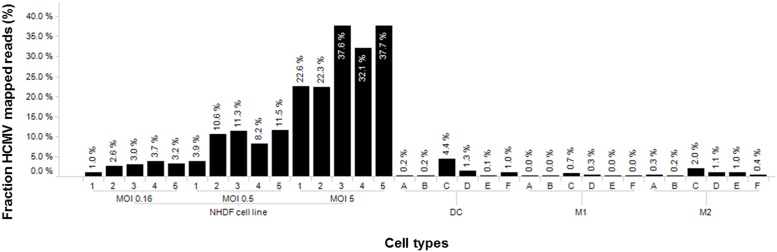
Percentage of mapped reads to the HCMV transcriptome in primary cell types. Six different blood donors (A-F) were processed to obtain monocytes which were differentiated to DCs, MΦ1 and MΦ2. Subsequently, these primary cell types were infected with TB40/E at MOI5. As a comparison, we infected NHDF cells (n = 3) at MOI 0.16, MOI 0.5 and MOI 5. Given in the top panel are the percentages of mapped reads on the HCMV genome for each cell type.

We isolated CD14^+^ monocytes from six independently processed blood donors (A-F in Fig 1) and differentiated these cells to pro-inflammatory DCs and MΦ1; and anti-inflammatory MΦ2. DCs were characterized as CD14^-^/CD163^-^/DCSIGN^++^ and upon LPS treatment they showed upregulation of CD80; MΦ1 were CD14^+^/CD163^-^/DCSIGN^+^ whereas MΦ2 showed a CD14^+^/CD163^+^/DCSIGN^+^ marker profile with poor upregulation of CD80 upon LPS treatment (S1 Fig).

Subsequently, these cell-types were infected with highly epithelial tropic TB40/E HCMV strain at MOI 5. In this study, the infection was carried out for 3 hours without subsequent inactivation of the remaining surface-bound virus e.g. with citrate buffer to avoid any damage or any changes in host transcriptome of the cells thereby attempting to keep the cell-virus interplay as authentic as possible. In contrast to fibroblasts where 100% of the cells could be infected, 3.11% (2.91–4.12%) of the DCs, 1.20% (1.01–1.36%) of the MΦ1 and 6.27% (3.5–8.16%) of the MΦ2 could be infected (S2 Fig).

Illumina RNA sequencing resulted in an average of almost 23.3 million (9.4–36.4 x 10^6^) sequences per sample covering both HCMV and host mRNA. In order to perform a comparative analysis of HCMV gene expression between fibroblasts and monocyte-derived cells, we argue that a more representative control is provided by infecting NHDF cells at a lower MOI thereby mimicking the actual percentage of HCMV reads in DCs and MΦs (Fig 1). Infection of DCs, MΦ1 and MΦ2 resulted in a median percentage of mapped reads of 0.63%, 0.04% and 0.67%, respectively. There was some variability in infectivity between the cells derived from different donors. We infected NHDF cells at three different MOIs and show that infection of NHDF fibroblasts at MOI 0.16 reflects better the percentage of reads mapped to the HCMV transcriptome (2.97%) in primary cells compared to MOI 0.5 (10.62%) and MOI 5 (32.12%) (Fig 1).

**Fig 2:**
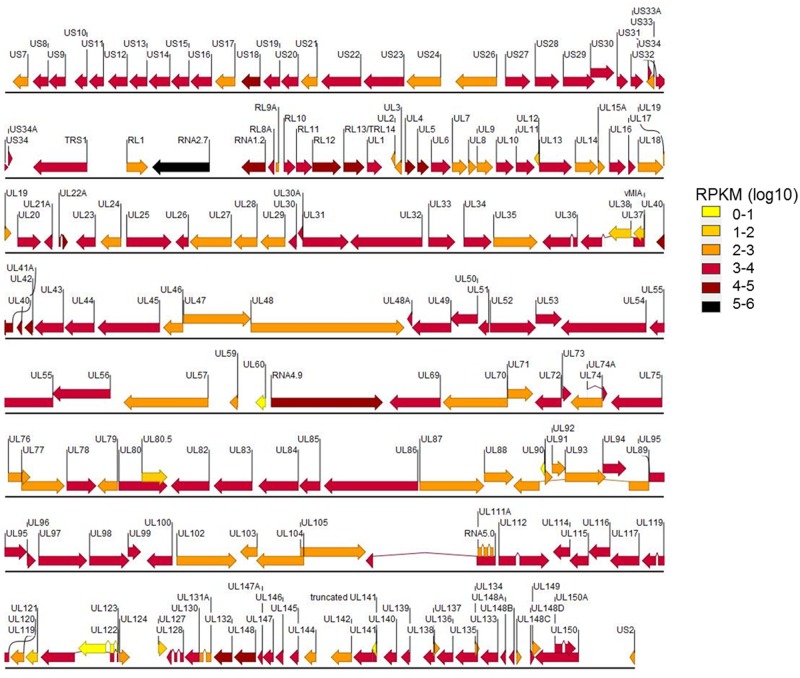
Expression levels of the HCMV genes transcriptome in fibroblasts (MOI 0.16). Log(10) of the RPKM values per gene were binned in six categories and mapped on the reference genome of TB40/E.

First, we evaluated the expression levels of each HCMV gene in NHDF cells at MOI 0.16. Expression levels are represented by the reads per kilobase of transcript per million (RPKM) value, which represents the number of mapped sequences per gene normalized for the gene length and the total amount of sequences from the experiment (accounting for experimental variability). We plotted the binned logs of these RPKM values on the HCMV genome annotation (acc.no. EF999921) used for this study (Fig 2).

The highest expressing genes in this study during lytic infection at low MOI in NHDF fibroblasts were RNA4.9, RNA2.7, RNA1.2, UL22A, US18, RL12, RL13-RL14, UL4, UL5, UL40, UL41A, UL42, UL132 and UL148. On the other end of the spectrum UL12, UL38, UL80.5, UL121, UL123, UL127, truncated UL141, and vMIA are only expressed at low levels (10–100). In contrast, no reads were mapped for UL60 and UL90 in this study.

### Differentially regulated genes and gene clusters in primary cell types

**Fig 3:**
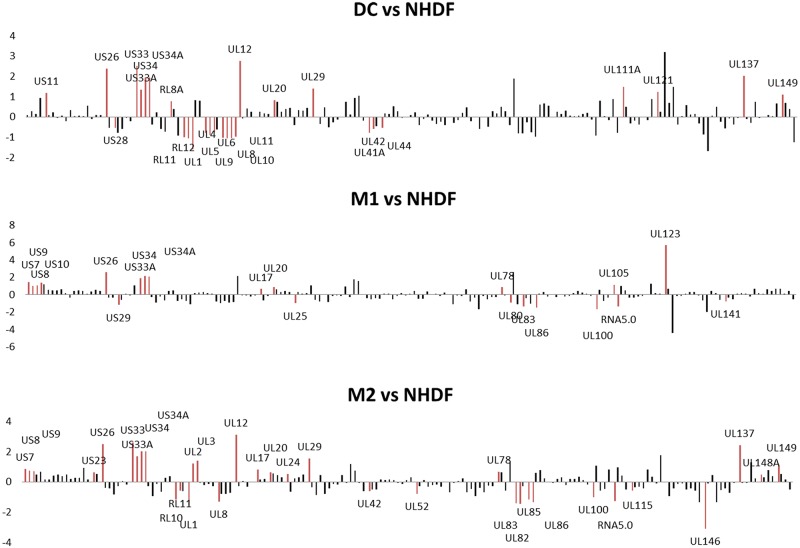
Differential regulation of HCMV gene expression in monocyte-derived DCs, MΦ1 and MΦ2 (MOI5) compared to lytic infection in NHDF fibroblasts (MOI0.16). On the graph is the fold increase or decrease (reflecting genes which are expressed higher/lower in fibroblasts than in primary cell types). Red bars represent significant changes in gene expression between the indicated cell type and NHDF fibroblasts (adjusted p<0.05), black bars indicate modulated genes which did not reach the significance threshold (adjusted p≥0.05).

Next, we compared HCMV gene expression in monocyte-derived DCs, MΦ1 and MΦ2 with HCMV gene expression in NHDF cells (Fig 3). In general, the regulation of the US region was striking in primary cell types. Especially US26, US33 (not in MΦ1), US33A, US34 and US34A were found to be expressed at higher levels in primary cells (p<0.01). Also in the US region, US7-US9, US10 (only significant in MΦ1), US11 (only significant in DCs), US23 (only significant in MΦ2) and US23 (only significant in MΦ2) were expressed to higher levels in primary cells. Striking was the significantly lower expression of US28 (in DCs) and US29 (in MΦ2) compared to NHDF cells.

In particular in DCs, the regulation of HCMV genes was clustered. RL11-RL12-RL13, UL41A-UL42 and (UL1)UL4-UL11 were present in lower amounts in primary cells while, besides the aforementioned genes in the US region, UL120-UL121 and UL148D-UL149 were found to be expressed higher. In MΦ1, except for the US region, this clustering was not as clear. Individual genes such as UL25, UL100, UL141, RNA5.0, UL80, UL83 and UL86 were detected at higher expression levels in contrast with the lower expression of UL17, UL20, UL78, UL105 and UL123. UL80, UL83 and UL86 may be part of a cluster. In MΦ2, RL10-RL11 and a cluster from UL82 to UL86 (p>0.05 for UL84) as well as individual genes such as RNA5.0, UL1, UL8, UL42, UL52, UL100, UL115 and UL146 were found to be lower expressed than in NHDF fibroblasts. Also genes in MΦ2 were more difficult to cluster, only UL2-UL2 and UL148A-UL149 were significantly higher expressed neighboring genes in addition to individual genes as UL12, UL17, UL20, UL29, UL78 and UL137.

In a head to head comparison between the different primary cell types, the only significant difference were the lower amounts of UL4-UL5, US7 and US9 in DCs compared to MΦ1. This suggests that the virus regulates its gene expression similarly in both macrophage subtypes, despite their different inflammatory character and infectability.

### Functional analysis of differentially regulated genes

Each HCMV gene has a specific function during the lytic or latent part of the viral life cycle. By annotating each gene with its function [40], we were able to explore if any functional groups were significantly enriched (S3 Fig). This means that the p-values of each functional group were compared between two cell types. The enrichment of a functional groups can be driven either by the differential expression of many genes with p-values between 0.05 and 0.01; or by a small number of highly significant genes (p<0.001).

**Fig 4:**
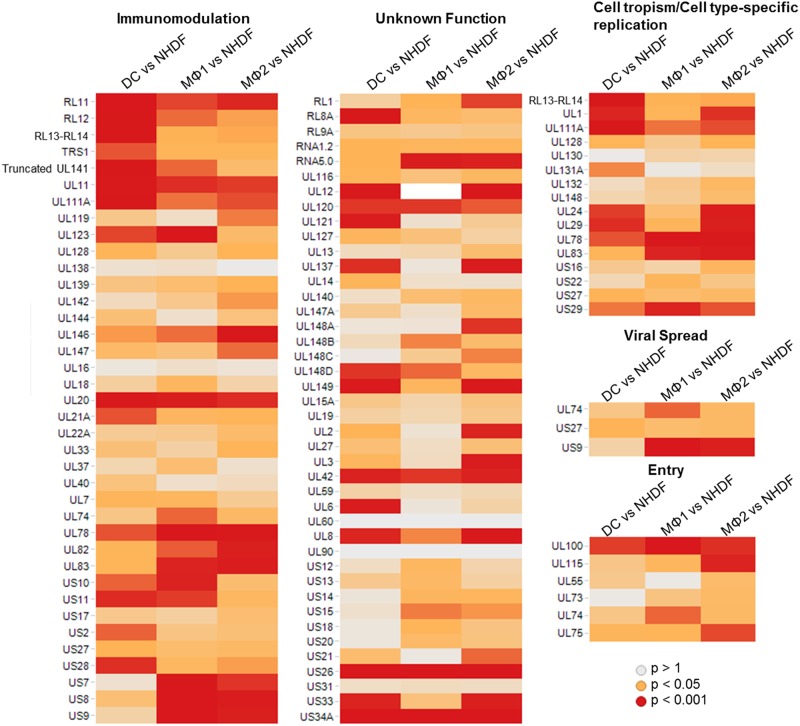
Enrichment of functional groups in DCs, MΦ1 and MΦ2 compared to NHDF. Genes involved in cell tropism, viral spread and immunomodulation are shown to be the most enriched groups in DCs, MΦ2 and MΦ1. Given are the p-vales of each individual gene.

This pathway analysis showed that in DCs and MΦ1, compared to NHDF fibroblasts, the most affected functional group consisted of genes involved in immunomodulation. In DCs, the second and third functional groups which were enriched contained genes which have not been annotated with a function or genes involved in cell tropism and cell-dependent replication, respectively. In MΦ1, genes with a function in viral spread and entry contributed to the enrichment of the similarly named functional classes. Finally, in MΦ2 only two functional groups were significantly affected, being genes involved in cell tropism and cell-specific replication; and genes involved in viral spread (Fig 4).

The group of immune-modulatory genes is the largest (38 tested genes) and the presence of many significant p-values (p<0.05) demonstrates the importance of the regulation of genes in this group in all examined monocyte-derived cells. In DCs, RL11-RL13, UL141, UL11, UL111A and UL20 were the most important differentially regulated genes involved in immunomodulation (p<0.01). To a lesser extent (0.01<p<0.05), TRS1, UL123, UL21A, UL78, US10-US11, US2 and US28 were contributing to the enrichment of this functional group. In MΦ1, functional analysis also revealed a shift in immunomodulatory genes, but this was driven by slightly different genes (based on significance) i.e. UL123, UL20, UL78, UL83, US10, US7, US8 and US9 (p<0.01) and to a lesser extend RL11-RL12, UL141, UL11, UL111A, UL146, UL74, UL82 and US11 (0.01<p<0.05).

In both DCs and MΦ2, the second most significant functional annotation is comprised of genes with a function in cell tropism and cell-specific replication. This was due to the modulation of different genes in DCs and MΦ2. In DCs, the p-values of RL13, UL1 and UL111A were the main contributors whereas in MΦ2 the identification of this pathway was driven by UL24, UL29, UL78 and UL83 (p<0.01). In MΦ1 and MΦ2, viral spread was also found to be an enriched functional group mainly influenced by the highly significant p-values of UL74 and US9. Finally, in MΦ1, UL110 and UL74 were the main contributors to the significant p-value of the functional group involved in entry processes.

**Fig 5:**
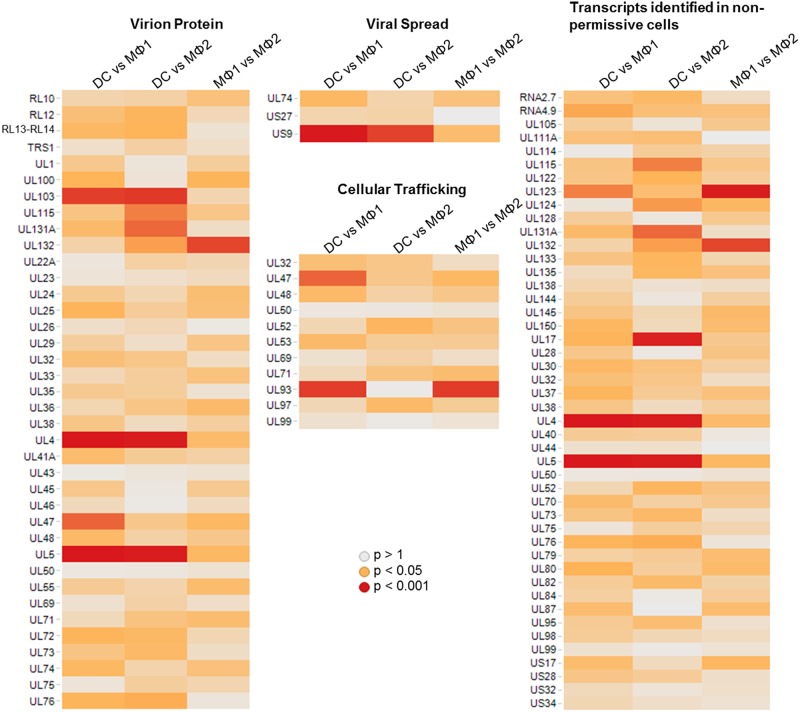
Enrichment of functional groups when comparing DCs with both types of macrophages and when comparing MΦ1 with MΦ2 macrophages. The HCMV was annotated and a mathematical model was used to determine which functional groups were significantly enriched in the differentially regulated genes. Based on this algorithm, genes involved in cell tropism, viral spread and immunomodulation are the most enriched groups in DCs, MΦ2 and MΦ1.

The same functional distributions were generated when primary cell types were compared to each other. Genes which were differentially regulated between DCs and macrophages mostly code for virion proteins (i.e. RL12, RL13.TRL14, US23, UL4-UL5, UL24-UL25, UL29, UL82-UL83, UL100 and UL115) which underlines the adaptability of the virion to the specific cell type where they are produced (Fig 5). When comparing DCs to MΦ1, US9 shows the highest significance in the group of factors involved in viral spread (Fig 5). Further, many differentially when comparing DCs to MΦ2 expressed genes were described in non-permissive cells and may be involved in latency (Fig 5). However, the relevance of most of these genes in lytic infection has not yet been established. Finally, when comparing DCs and MΦ2, genes contributing to cell tropism and cell-dependent replication such as UL132 and UL148 were responsible for the significance of this functional group. Between pro- and anti-inflammatory macrophage types, UL93 drives the significant enrichment of cellular trafficking factors (Fig 5).

## Discussion

The organization of regulatory motifs in different promotor regions on the HCMV genome shows the potential for HCMV to intricately regulate its transcription under specific circumstances [41]. This was recently confirmed with rat CMV (RCMV) which followed an alternative transcriptional profile in commonly used cell lines in the laboratory and cell types which are relevant *in vivo* [42].

Macrophages (MΦ) and dendritic cells (DCs) can be infected with HCMV *in vivo* [43–47] and *in vitro* [44, 48]. Both cell types play a role in latency and reactivation since their progenitors i.e. CD14^+^ monocytes and CD34^+^ cells support latency whilst terminal differentiation to DCs or MΦ results in reactivation [8, 18, 30, 49–52]. Because of the limited knowledge about transcription in these cell lines and their potentially vital role *in vivo*, we set up a next generation sequencing study to investigate the profile of the HCMV transcriptome in monocyte-derived pro-inflammatory MΦ1, anti-inflammatory MΦ2 and DC compared to lytic infection in NHDF fibroblasts.

We infected pro-inflammatory CD14^-^/CD163^-^/DCsign^+(hi)/^CD80^+(+LPS)^ monocyte-derived DCs, pro-inflammatory CD14^+^/CD163^-^/DCsign^+(lo)^/CD80^+(+LPS)^ MΦ1 and anti-inflammatory CD14^+^/CD163^+^/DCsign^+(lo)^/CD80^-(+LPS)^ MΦ2 [34, 53–62]. As reported before, MΦ2 were easier to infect than MΦ1 [44] resulting in respectively 6.27% (3.5–8.16%) and 1.2% (1.01–1.36%) infection. The infectivity of DCs was intermediate between MΦ1 and MΦ2 (3.11%, 2.91–4.12). These percentages were reflected in the number of mapped reads on the HCMV genome. A study by Bayer et al. [44] reports infection rates up to 75% in MΦ2; however in none of the donors used in this study we obtained infection rates above 10%. While in literature the polarization in macrophages is perceived distinct, the differences between these cell types is much more complex and subtle [58, 60, 63, 64]. It has been shown that a plethora of monocyte-derived MΦs exists under the influence of cytokines [65], but also under influence of FCS on its own, distinct MΦ subtypes are produced [66, 67]. Therefore, it is not unlikely to assume that small technical differences such as the method of monocyte isolation, plate type, the source of the cytokines, differences in the culture medium or the handling of the buffy coat pre-isolation have an impact on the type of monocyte-derived cell and thus the rate of infection [68, 69].

For the RNA sequencing analysis, we updated the sequence of the TB40/E-BAC4 (EF999921; 2008) with later gene annotations from KF297339 (2013). Of note, the complexity of the transcriptome may still increase as the clinical Merlin strain was recently annotated with hundreds of additional ORFs [26]. However, because genetic differences between strains may influence the transcriptome [23], we limited our analysis to the available TB40/E sequence information.

We used infection in NHDF fibroblasts as a reference cell type for lytic infection to compare HCMV gene expression in MΦ1, MΦ2 and DCs. It has been reported that MΦs support productive lytic infection characterized by the shedding of virus [44]. In agreement with these published results, all viral transcripts except UL60 and UL90, including late genes were detected in this study. We, and others, observe that the majority of the HCMV reads in NHDF, DCs, MΦ1 and MΦ2 is dedicated to RNA4.9, RNA2.7 (36% of all HCMV specific reads), RNA1.2, UL4, UL5 and UL22A transcripts [24]. These similarities between NHDF and the primary cell types suggest a lytic program and not a latency transcriptional profile as in CD34^+^ progenitor cells which only express a restricted set of transcripts and do not follow the immediate-early, early, late gene expression cascade [8, 70]. It has to be noted that the presence of late transcripts does not always rule out the presence of some degree of abortive infection as late virion-associated genes have been identified as well [71]. However, only one of these virion-associated genes (RL13) was of any significance in this study. Taken together, we conclude that in this study primary cells displayed a lytic transcriptional program.

In primary cell types, differentially regulated genes were often found in clusters suggesting that these clustered genes may have similar functions. In DCs, the downregulation of RL11-RL13-RL14-UL1 and UL4-UL11; and the upregulation of UL120-UL121, UL148D-UL149 and US33-US34A are striking. In MΦ2, RL11-RL12 and UL82-UL86 (UL84 not significant) were upregulated and besides UL148A-UL149 and UL2-UL3, very strong clustering was seen in the US region where US7-US9 and US33-US34A were upregulated. A very similar observation was made for MΦ1 but the downregulated cluster of UL80-UL86 was interrupted because the lower expression of UL81-UL82 and UL85 did not reach significance. Although the functions of many of the HCMV genes remain unknown, pathway analysis reveals that the functional group of immunomodulatory genes is significantly enriched in DCs, MΦ1 and to a lesser extend in MΦ2 compared to NHDF fibroblasts. These observations were made before in several focused studies which showed a profound effect of HCMV on the host immune-transcriptome and an active modulation of viral transcripts via viral proteins such as pp65 [31, 72–75] but recently also a transcriptomics study with RCMV showed an enrichment of immunomodulatory genes in relevant *in vivo* cell types [42]. Especially the regulation of the US region contributed to the enrichment of this functional group. Of note is that TB40/E-BAC4 used in this study misses US1-US6 compared to the wild type virus [32]. Future research with other strains such as Merlin may elucidate if US1-US6 are also part of this unique transcriptional program.

The US region was associated with immunomodulation before [72, 76–88]. More specifically, differentially expressed genes such as US8, US10 and US11 have a role in the modulation of MHCI molecules [76–84, 89], US9 was reported to be involved in the evasion of NK activation [90], US28 interferes with cyto- and/or chemokine production or responsiveness [72, 87, 88] and based on sequence similarity the miRNA regulated US7 [91] and the largely unknown US9 are predicted to have similar roles [92]. Other studies such as a study using superSAGE describing the transcriptome in DCs indicated the importance of immunomodulatory genes in HCMV infection of DCs [31]. Further, also in *in vivo* cell types infected with RCMV it was suggested that the differential expression of immunomodulatory genes is a unique way for the virus to evade the immune system during infection [42]. Also, several groups evaluated the host’s transcriptome in monocytes which, under the influence of HCMV, are driven towards a MΦ1 phenotype. These groups found that in the host’s transcriptome, most genes that were differentially regulated were involved in anti-viral responses, inflammatory response, viral spread and apoptosis.

The US region is not fully annotated with functions and some of the most significantly regulated genes in this study such as US26 and US33-34A have unknown functions [24]. US34 was identified before as an abundantly expressed transcript in DCs [31] and US34A was identified as a SUMOylation target [93]The relevance of these observations is not clear but based on this study a role in immunomodulation would not be surprising.

Next to genes involved in immunomodulation, we found that also factors involved in cell tropism and cell-specific replication were enriched, especially in DCs and MΦ2 compared to NHDF fibroblasts. The genes responsible for this enrichment are different for both cell types i.e. RL13-RL14, UL1 and UL111A in DCs versus UL24, UL29, UL78 and UL83 in MΦ2. The differential regulation of cell-tropism factors but also of viral spread factors (driven by UL74, US27 and US9) suggests that the virion may adapt to optimally infect surrounding cells of the same type as previous research already suggested [27, 94].

In addition, there are also discrete differences when comparing the different primary cell types head to head. Between DCs and MΦ1, there were four genes (UL4, UL5, US7 and US9) significantly lower expressed in DCs. While US7 and US9 only have proposed roles, UL4 and UL5 are virion proteins and their differential regulation may once more suggest the adaptability of the virion when infecting different cell types. Despite their different inflammatory character and infectability no genes were found to be differentially expressed (p<0.05) when comparing MΦ1 and MΦ2 or when comparing DCs and MΦ2. In a previous study investigating HCMV infection in both MΦ subtypes, it was reported that IE1-2 (UL122-123), pp65 (UL83) and pp150 (UL32) were more abundantly present in MΦ2 [44], however in this study this difference did not reach significance. Perhaps, if a sequencing study was carried out on different time points, these differences could reach significance.

The HCMV genome is foreseen of several regulatory motifs and can replicate in a variety of cell types suggesting that in each of these cell types, a specific transcriptional program is followed [42, 95]. This HCMV study is the first transcriptomics study which compares gene expression in commonly used NHDF fibroblasts and DCs, MΦ1 and MΦ2, all cell types which are considered relevant for *in vivo* viral spread. By using pathway analysis, we show that compared to fibroblasts, a unique transcriptional profile is observed in primary cells in which genes involved in immunomodulation genes, cell tropism and replication, viral spread and virion proteins are enriched. While the differential regulation of these specific pathways is still consistent with lytic infection, it resembles more closely a lytic low level persistent infection with careful adaptations to the cellular environment rather than with a fulminant lytic infection as observed in fibroblasts. These observations not only show the adaptability of the virus, but also explain any discrepancies between what may happen *in vivo* and what is observed in the laboratory.

As in the RCMV study, many questions remain unanswered. We report a number of genes that are strongly upregulated in the US region, but defined functions for these genes have not been described and functional research is needed to delineate their actual role in the viral life cycle. Especially, the high expression of the US33-US34 cluster warrants further research as it is the first time that alterations in gene expression levels are observed for these genes. It would be interesting to attribute these genes with critical functions in primary cells. In addition, it is unclear whether cellular factors in immune cells such as DC and MΦs push the virus into the production of specific transcripts or if it is the virus that manipulates the cell by producing transcripts that aid with immune-evasion. Most likely it is a combination of both. Several groups evaluated the host’s transcriptome in monocytes which, under the influence of HCMV, are driven towards a MΦ1 phenotype. These groups found that in the host’s transcriptome, most genes that were differentially regulated were involved in anti-viral responses, inflammatory response, viral spread and apoptosis which is consistent with the differential regulation of viral genes involved in these processes reported in this study [96–98]. Yet, it is an intriguing observation that MΦ1 and MΦ2, which display distinct polarization, show a similar viral transcription profiles. Also, it may be warranted to dedicate future research to investigate regular and short lived HCMV transcripts using specialized methods preferably in a kinetic time course set up [99]. Finally, since expression levels of transcripts are not always reflected in actual protein expression profiles, it would be interesting to correlate the findings presented here to proteomics data once these data are available.

## Supporting Information

S1 Fig Surface marker analysis of monocyte-derived cell types. Monocytes were seeded and differentiated to MΦ1, MΦ2 and DCs or left untreated. After 7 days, the cells were characterized using FACS. The boxplots are based on 11 different and independently processed donors. The boxes represent all counts within the 25^th^ and 75^th^ percentile; error bars show the 10^th^ and 90^th^ percentile. All values outside the 10^th^ and 90^th^ percentiles are considered outliers (•), the mean is indicated as a horizontal line. A one way ANOVA (Holm-Sidak method) was used to test for significant differences between cell types. (**Panel A**) CD14 surface expression was significantly different (p>0,05) between all cell types (Mo vs MΦ1, Mo vs MΦ2, Mo vs DC; MΦ1 vs DC; MΦ2 vs DC), except between MΦ1 and MΦ2 (p>0.05). (**Panel B**) DC marker DCSign was expressed significantly higher on DCs and all other cell types (p<0.05; Mo vs DC, MΦ1 vs DC, MΦ2 vs DC). DCSign was also expressed more on both types of MΦ compared to monocytes (p<0.05). No statistical difference was observed between both types of macrophages (p>0.05). (**Panel C**) CD163 was only expressed on MΦ2 and poorly on all other cell types (p<0.05; MΦ2 vs Mo, MΦ2 vs MΦ1, MΦ2 vs DC). (**Panel D**) To assess CD80 response, all cell types were challenged with 500ng/ml LPS for 24h. MΦ2 macrophages showed a significantly lower response in C80 upregulation compared with the other cell types (p<0.05; MΦ2 vs Mo, MΦ2 vs MΦ1, MΦ2 vs DC). CD80 expression between all other cell types was comparable (p>0.05).
(TIF)

S2 Fig A representative image (donor A) of the number of IE expressing cells in TB40/E and mock (UV-inactivated TB40/E) infected primary cell types. In each representative image, we indicated the average number and the range of IE expressing cells for three independently processed donors.
(TIF)

S3 Fig Distribution of functions of DCs, MΦ1 and MΦ2 compared to NHDF fibroblasts and of primary cells compared to each other. The most enriched functions are represented by the highest bars. The cut off to determine the most significantly enriched functions was based on the point where the box plot curves flatten out (marked by a red box).
(TIF)
