# Supplementary figures and images for: HCMV Displays a Unique Transcriptome of Immunomodulatory Genes in Primary Monocyte-Derived Cell Types
Source: PLoS One. 2016 Oct 19;11(10):e0164843. doi: 10.1371/journal.pone.0164843 (PMC5070835; doi:10.1371/journal.pone.0164843)

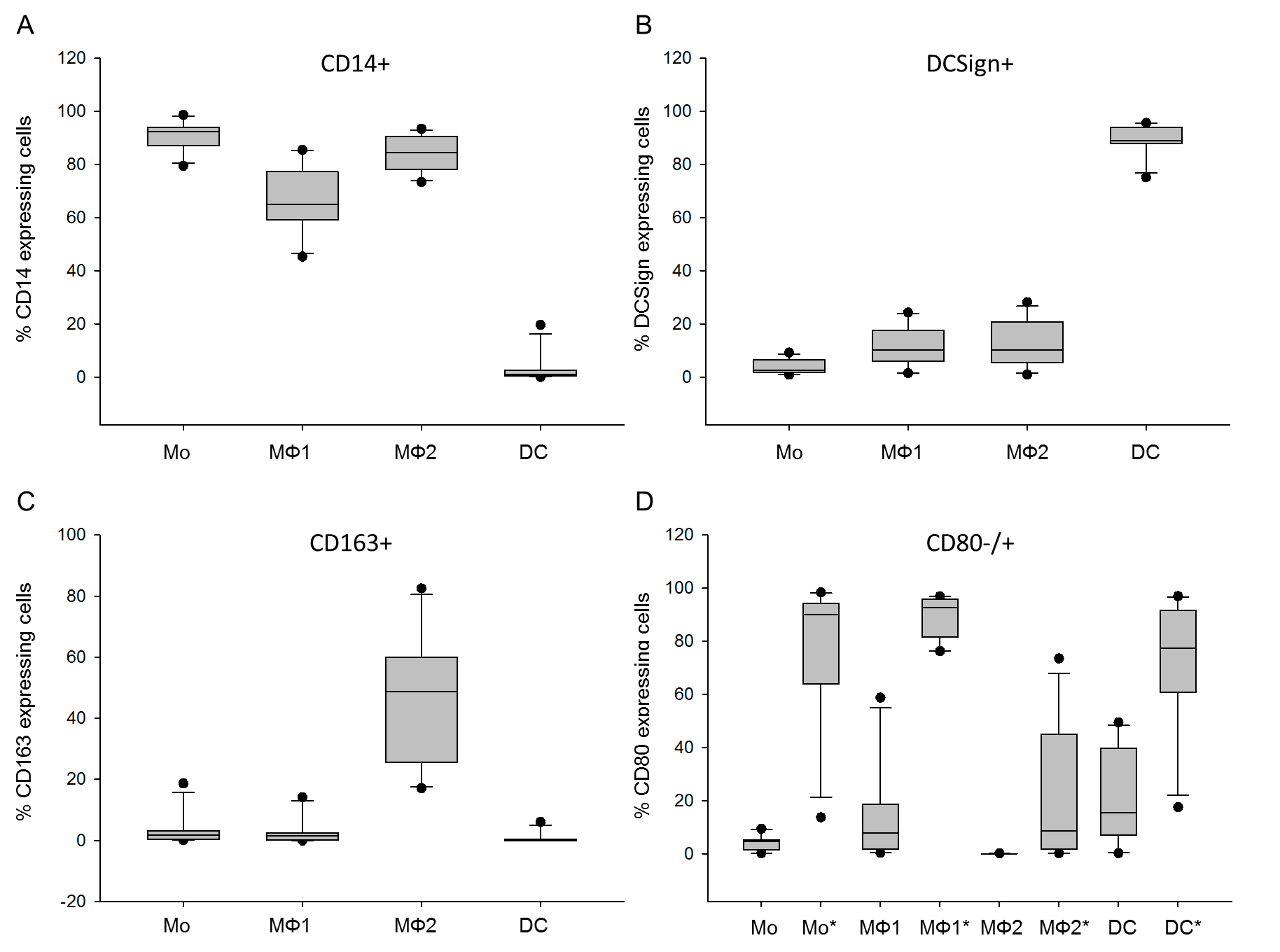

Supplement: S1 Fig — Monocytes were seeded and differentiated to MΦ1, MΦ2 and DCs or left untreated. After 7 days, the cells were characterized using FACS. The boxplots are based on 11 different and independently processed donors. The boxes represent all counts within the 25th and 75th percentile; error bars show the 10th and 90th percentile. All values outside the 10th and 90th percentiles are considered outliers (•), the mean is indicated as a horizontal line. A one way ANOVA (Holm-Sidak method) was used to test for significant differences between cell types. (Panel A) CD14 surface expression was significantly different (p>0,05) between all cell types (Mo vs MΦ1, Mo vs MΦ2, Mo vs DC; MΦ1 vs DC; MΦ2 vs DC), except between MΦ1 and MΦ2 (p>0.05). (Panel B) DC marker DCSign was expressed significantly higher on DCs and all other cell types (p<0.05; Mo vs DC, MΦ1 vs DC, MΦ2 vs DC). DCSign was also expressed more on both types of MΦ compared to monocytes (p<0.05). No statistical difference was observed between both types of macrophages (p>0.05). (Panel C) CD163 was only expressed on MΦ2 and poorly on all other cell types (p<0.05; MΦ2 vs Mo, MΦ2 vs MΦ1, MΦ2 vs DC). (Panel D) To assess CD80 response, all cell types were challenged with 500ng/ml LPS for 24h. MΦ2 macrophages showed a significantly lower response in C80 upregulation compared with the other cell types (p<0.05; MΦ2 vs Mo, MΦ2 vs MΦ1, MΦ2 vs DC). CD80 expression between all other cell types was comparable (p>0.05). (TIF) [file pone.0164843.s001.tif]

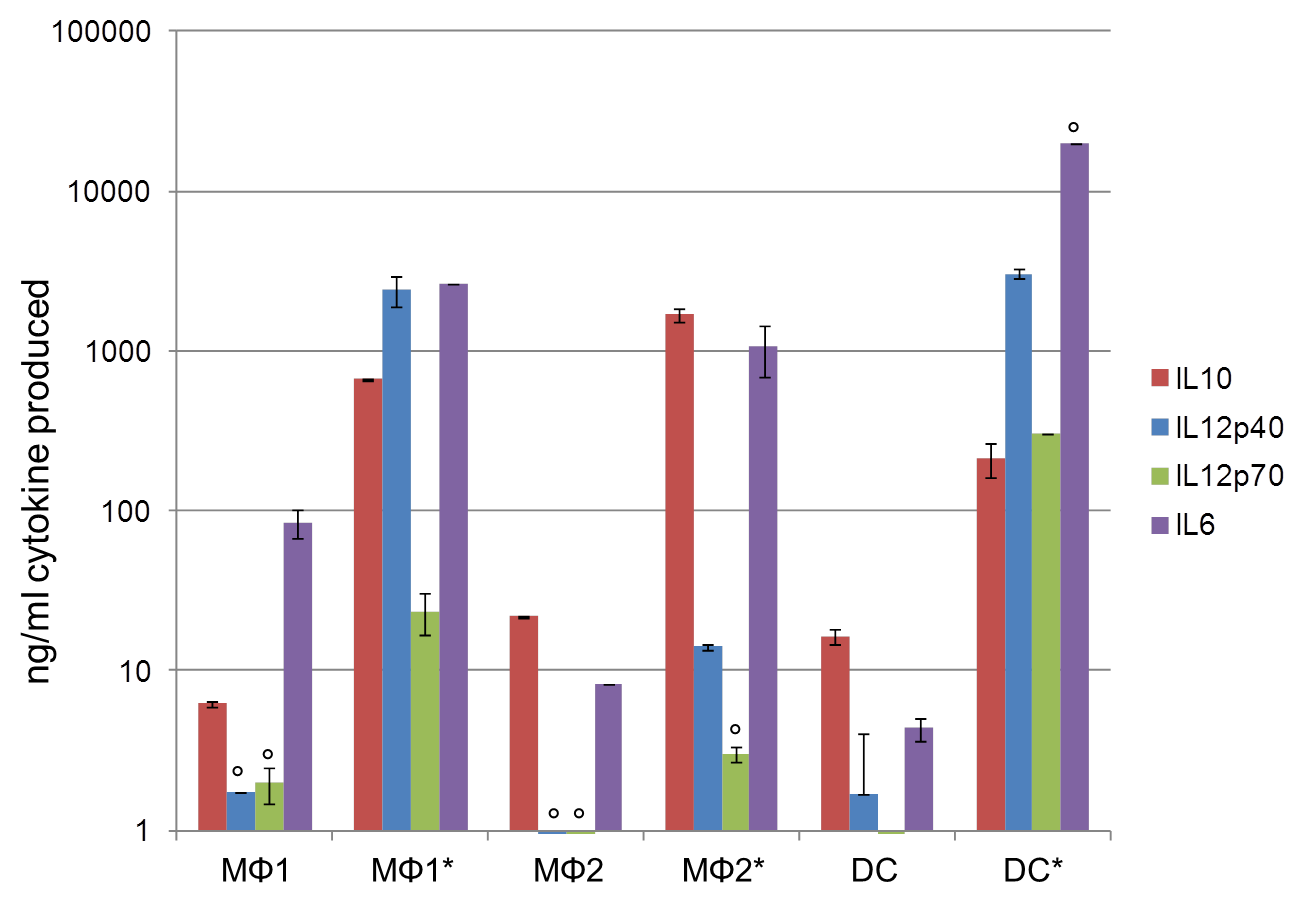

Supplement: S2 Fig — In each representative image, we indicated the average number and the range of IE expressing cells for three independently processed donors. (TIF) [file pone.0164843.s002.tif]

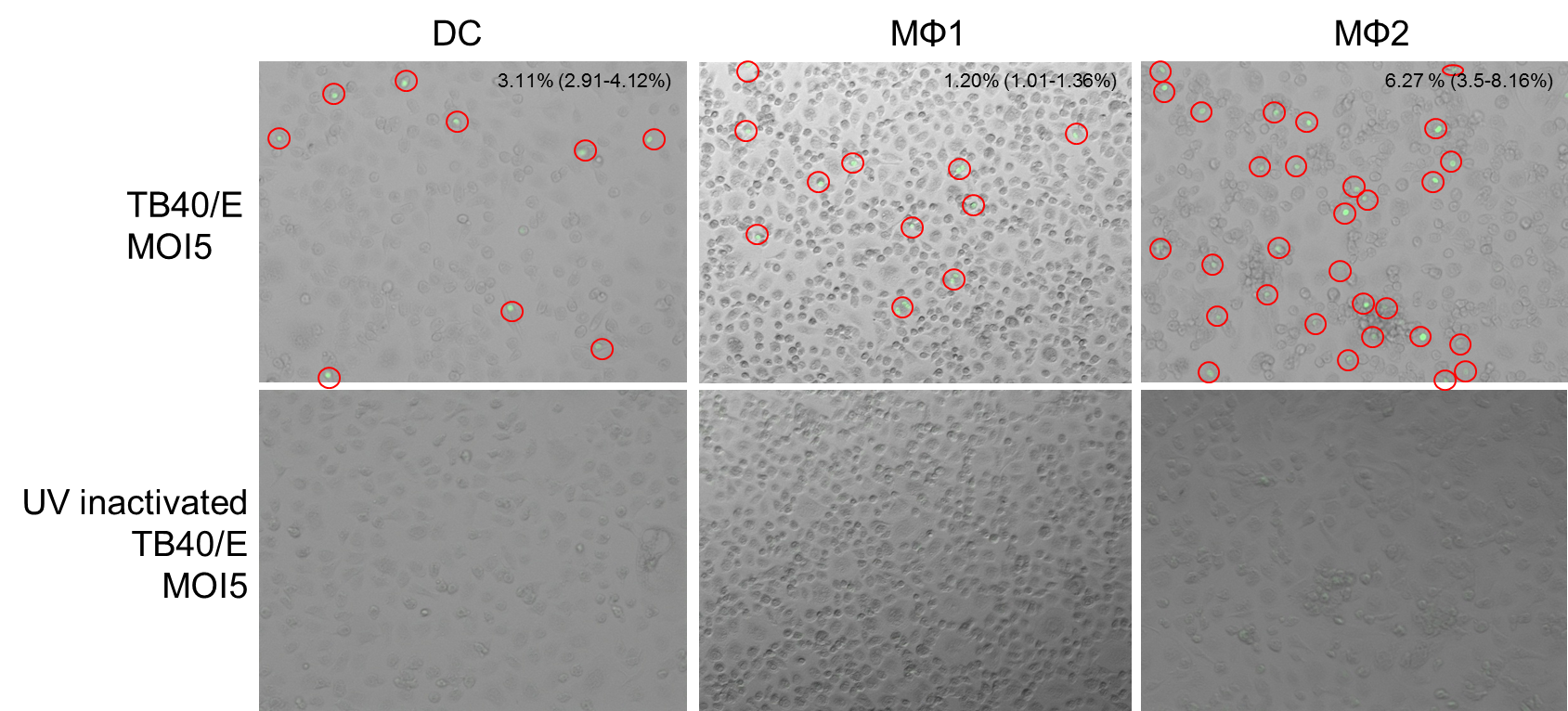

Supplement: S3 Fig — The most enriched functions are represented by the highest bars. The cut off to determine the most significantly enriched functions was based on the point where the box plot curves flatten out (marked by a red box). (TIF) [file pone.0164843.s003.tif]
